# mirEX 2.0 - an integrated environment for expression profiling of plant microRNAs

**DOI:** 10.1186/s12870-015-0533-2

**Published:** 2015-06-16

**Authors:** Andrzej Zielezinski, Jakub Dolata, Sylwia Alaba, Katarzyna Kruszka, Andrzej Pacak, Aleksandra Swida-Barteczka, Katarzyna Knop, Agata Stepien, Dawid Bielewicz, Halina Pietrykowska, Izabela Sierocka, Lukasz Sobkowiak, Alicja Lakomiak, Artur Jarmolowski, Zofia Szweykowska-Kulinska, Wojciech M. Karlowski

**Affiliations:** Department of Computational Biology, Institute of Molecular Biology and Biotechnology, Faculty of Biology, Adam Mickiewicz University, Umultowska 89, 61-614 Poznan, Poland; Department of Gene Expression, Institute of Molecular Biology and Biotechnology, Faculty of Biology, Adam Mickiewicz University, Umultowska 89, 61-614 Poznan, Poland

**Keywords:** microRNA, Gene expression, Database, *Arabidopsis thaliana*, *Hordeum vulgare*, *Pellia endiviifolia*

## Abstract

**Background:**

MicroRNAs are the key post-transcriptional regulators of gene expression in development and stress responses. Thus, precisely quantifying the level of each particular microRNA is of utmost importance when studying the biology of any organism.

**Description:**

The mirEX 2.0 web portal (http://www.combio.pl/mirex) provides a comprehensive platform for the exploration of microRNA expression data based on quantitative Real Time PCR and NGS sequencing experiments, covering various developmental stages, from wild-type to mutant plants. The portal includes mature and pri-miRNA expression levels detected in three plant species (*Arabidopsis thaliana*, *Hordeum vulgare* and *Pellia endiviifolia*), and in *A. thaliana* miRNA biogenesis pathway mutants. In total, the database contains information about the expression of 461 miRNAs representing 268 families. The data can be explored through the use of advanced web tools, including (i) a graphical query builder system allowing a combination of any given species, developmental stages and tissues, (ii) a modular presentation of the results in the form of thematic windows, and (iii) a number of user-friendly utilities such as a community-building discussion system and extensive tutorial documentation (e.g., tooltips, exemplary videos and presentations). All data contained within the mirEX 2.0 database can be downloaded for use in further applications in a context-based way from the result windows or from a dedicated web page.

**Conclusions:**

The mirEX 2.0 portal provides the plant research community with easily accessible data and powerful tools for application in multi-conditioned analyses of miRNA expression from important plant species in different biological and developmental backgrounds.

**Electronic supplementary material:**

The online version of this article (doi:10.1186/s12870-015-0533-2) contains supplementary material, which is available to authorized users.

## Background

MicroRNAs are short, predominately 20–22 nucleotide small RNAs that function as versatile gene expression regulators in development and stress responses. Plant miRNAs are produced from primary transcripts (pri-miRNAs) that form a miRNA/miRNA*-containing stem-loop structure. They are processed by the DICER-LIKE 1 enzyme (DCL1) [1] which forms a microprocessing complex with SERRATE (SE) [2] and HYPONASTIC LEAVES 1 (HYL1) [3] proteins. Another ssRNA-binding protein, TOUGH (TGH), plays an important role in the efficient recruitment of the DCL1-HYL1-SE complex to pri-miRNA [4]. Additionally, SERRATE interacts with the Cap-Binding Complex (CBC), which is a heterodimer of CAP BINDING PROTEIN 20 (CBP20) and CAP BINDING PROTEIN 80 (CBP80) [5]. These interactions mainly ensure the proper efficiency of pri-miRNA processing [6, 7]. The miRNA/miRNA* duplex is exported to the cytoplasm with the help of the HASTY1 (HST1) protein which is involved in the nuclear export of microRNAs [8]. In the cytoplasm the miRNA guide strand is selectively loaded into ARGONAUTE1 (AGO1) to form the RNA-Induced Silencing Complex (RISC) responsible for mRNA slicing [9]. HUA ENHANCER 1 (HUA1) methylates miRNA/miRNA* duplexes probably before they are loaded onto the AGO1 protein and renders microRNAs more stable [10–12]. The miRNA-loaded RISC directs the posttranscriptional silencing of the complementary target mRNA. The target mRNA is predominantly cleaved or its translation is repressed [13–16].

Genes that encode transcripts that are processed to the same or similar mature microRNA species are grouped into families. In many cases it is only possible to observe the expression of all family members as a group rather than that of individual members by using Northern hybridization, RT-qPCR or sequencing approaches. However, individual members of a given microRNA family may be expressed in different developmental stages or in response to various biotic/abiotic stimuli [17–20]. In addition, most of the plant miRNA genes are family- or species-specific, and unlike the conserved genes, young miRNAs are often weakly expressed and imprecisely processed. In these cases the analysis of microRNA primary transcripts (pri-miRNA) expression can be informative and point to an individual family member gene that undergoes expression changes.

Recently, genome-wide analyses from several plant species have revealed that variation in miRNA expression and processing has an impact on micro RNA unique functionality [21, 22].

At present, the amount of expression data produced by sequencing technologies in many cases overbalances the potential of data exploration tools. Therefore, it is of outmost importance to create user-friendly and powerful tools that will allow for new hypothesis testing and application of novel approaches for data exploration. Although a few databases include expression information of miRNAs [23–26], their coverage is quite limited and fail to integrate most of the high-throughput experimental results. Additionally, in most cases the available repositories do not provide ability to simultaneously compare expression levels between various microRNA genes in diverse organs and developmental stages.

We have developed mirEX – a portal dedicated to comparative data mining of microRNA expression information in plants, from the studies of wild-type and mutant species, covering various developmental stages and including data from RT-qPCR, northern blot and NGS experiments. The portal contains data, which present *MIR* gene expression at the level of transcript (pri-miRNA) and mature miRNA [27]. Currently, the enhanced system (mirEX 2.0; see Additional file 1: Table S1 for a full list of updated features in current release of mirEX portal) allows for comparative miRNA data exploration with a simple, graphical querying system as well as with advanced visualization and data presentation tools within plant species representing three major groups: dicots (*Arabidopsis thaliana*), monocots (*Hordeum vulgare*), and bryophytes (*Pellia endiviifolia)*. Arabidopsis, the well-established reference plant model, is represented by wild-type plants as well as by several miRNA-biogenesis mutants. Barley represents one of the most agronomically important plants that can serve, in many aspects, as a model organism for other closely related grass species. *P. endiviifolia* belongs to liverworts, i.e., plants that are most probably the basal group of land invaders [28]. The mirEX 2.0 database is the first resource to provide comparative data on the microtranscriptome of these land-pioneering organisms.

The mirEX 2.0 portal is dedicated to researchers working on specific microRNA functions and expression profiles of entire microRNA family members during a particular organ/developmental stage or on microRNA biogenesis and evolution.

## Construction and content

### Developmental stages and tissues

The Arabidopsis developmental phases were classified according to Boyes et al. [29] and include four principal growth stages: leaf development (stage 1), rosette growth (stage 3), inflorescence emergence (stage 5), and flowering (stage 6). For each stage, total RNA was isolated from whole seedlings (stage 1), or in the case of older plants from rosette leaves, and in some cases from other organs, e.g., the stem, inflorescence and silique. Additionally, the levels of pri-miRNA transcripts were measured in dormant seeds. The barley developmental stages were classified according to Zadoks et al. [30], and total RNA was isolated from whole plants collected in five major growth points: 1-week-old (code 11) and 2-week-old seedlings (code 13), the beginning of tillering (code 20–21), stem elongation (code 32–36), and milk development in kernel (code 75–77). *Pellia endiviifolia* is represented by two types of plants: female and male thalli collected as environmental samples producing archegonia and antheridia, respectively, and female and male thalli grown *in vitro* without sex organs [31].

### Plant mutants

The mirEX 2.0 portal provides measurements of pri-miRNA expression levels in four arabidopsis mutants: the double mutant of the CBC subunits (*cbp20*x*cbp80*) [6], dicer-like 1 (*dcl1-7*) [1], hyponastic leaves (*hyl1-2*) [32], and *serrate* (*se-1*) (3). The selected mutants represent the key components of the microRNA biogenesis pathway in the plants [33] and show several specific molecular and phenotypic features. In all cases the reduction in the functionality of these proteins results in a decrease in the processing efficiency of miRNA precursor transcripts (pri- and pre-miRNA) and in a decrease in the amount of mature molecules [1, 6, 7, 32, 34]. Such dramatic changes in the microRNA metabolism induce severe abnormalities in both the structure and function of the mutant plants.

The *dcl1-7* plants contain a point mutation in the DCL1 RNA-helicase domain that is manifested in small leaves with an altered shape, delayed bolting and floral transition. Additionally, the mutants show reduced pollen production and disturbed ovule development, causing female sterility [35, 36]. The *hyl1-2* plants are much shorter than the wild-type with characteristic narrow and hyponastic leaves. The roots show reduced gravitropic response and plagiotropic growth. The lack of the HYL1 protein leads to delayed flowering – the flowers are smaller and fertility is reduced, the siliques are short and twisted [37]. Additionally, *hyl1* mutants show hypersensitivity to several phytohormones: abscisic acid (ABA), auxin and cytokinin [37], as well as enhanced response to abiotic stress [38]. The leaves of the *serrate* mutant (*se-1*) are notched (serrated) and do not curl abaxially. The plants have fewer juvenile leaves and show delayed floral initiation and extended flowering time. The flowers of the *se-1* mutant show extra sepals and petals [39, 40]. Similarly to *hyl1-2*, *se-1* shows hypersensitivity to ABA and amplified response to abiotic stress [38]. T-DNA insertions in genes encoding the subunits (CBP20 and CBP80) of CBC induce a similar phenotypic response as the *se-1* mutation. The rosette leaves of the mutant plants have serrated margins and show retarded growth [41]. Both proteins have been reported to be important in the ABA transduction pathway and their lack results in reduced wilting during drought [41–45] but decreased tolerance for high salt concentrations during germination [46].

### Expression data

The data available in the mirEX 2.0 portal cover the expression of 461 miRNAs representing 268 families, integrate the profiles for pri-miRNAs measured by RT-qPCR, and contain information about mature miRNA levels extracted from Next-Generation Sequencing (NGS) as well as Northern blot hybridization experiments. The data set of *Arabidopsis thaliana* miRNA sequences includes 299 pri-miRNAs representing 194 families. The expression data for microRNA primary transcripts from two-row spring barley *Hordeum vulgare* (cultivar Rolap [47, 48]) and liverwort *Pellia endiviifolia* [49, 50] contain 140 (57 families) and 22 (17 families) pri-miRNA sequences, respectively. The names for all of the miRNA sequences included in the current database were retrieved from miRBase version 21 [51].

For each developmental stage and organ studied we provide the expression data obtained by RT-qPCR representing pri-miRNA transcript levels. For the quantitative PCR experiments we include a number of reference genes: elongation factor 1-alpha (EF1-alpha, TAIR locus: *At1g07930*), glyceraldehyde-3-phosphate dehydrogenase C subunit 1 (gapdh, TAIR locus: *At1g13440*), polyubiquitin 10 (ubq10, TAIR locus: *At4g05320*) for arabidopsis [52], ADP-ribosylation factor 1-like for barley (GenBank AC: AJ508228.2) [53], and *ACTIN1* for *Pellia* (GenBank AC: DQ100290) [54]. The portal also includes data for mature miRNA expression based on NGS experiments. Incorporation of the various expression data types sparked the development of new exploration tools and substantially enhanced the comparative potential of the mirEX 2.0 database. High-throughput sequencing expression profiling is based on 31 independent sequencing samples, from which 21 were generated by our group.

The content of the database is constantly being updated and the most recent number of records as well as documentation regarding employed methods and procedures can be found on the mirEX 2.0 web page. All data that have been used to create the mirEX 2.0 portal can be downloaded from the web page and from interactive windows, and can be used in subsequent data-mining applications.

### Web implementation

The database was implemented in mySQL 5.5 [55] and the website is implemented in the Django web framework [56]. The service runs on an Apache server with a LINUX operating system. The front-end layout was created in HTML5 and CSS3 technologies on a Twitter Bootstrap 3.2.0 framework [57] to automatically adjust and adapt to different device screen sizes. The dynamically updating customizable windows containing the results are created using JavaScript (with the jQuery library) and AJAX technologies. The results are generated and presented to the user using several web-based libraries, such as d3.js [58], DataTables [59], as well as back-end solutions, such as R [60] and SciPy library for Python [61].

## Utility and discussion

### Data access

**Fig. 1:**
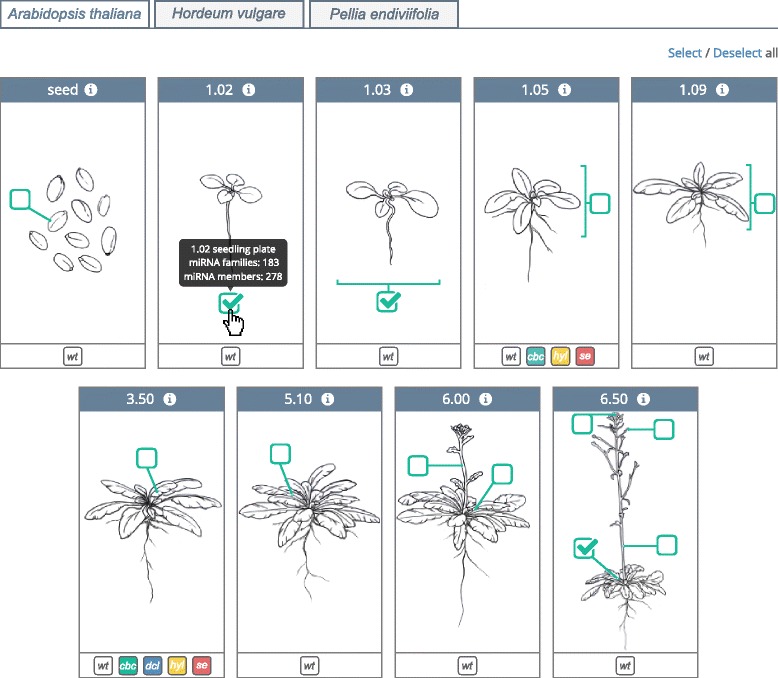
mirEX 2.0 graphical query builder window for *A. thaliana* tissue and developmental stage selection. The bottom part of each box includes links to expression data from mutant plants

The mirEX 2.0 portal offers a modern and graphical interface that allows to query and explore all of the information contained in it. The data can be accessed by searching for a particular microRNA name or by browsing the database content. We employ a simple, two-step querying system despite the vast amount and variability of data contained in the mirEX 2.0 database. The first step in the data selection process is provided in the form of a graphical query builder (Fig. 1) to supply the functionality for selecting any combination of species, developmental stages and microRNAs.

Selecting a mutant ID (Fig. 1) results in the presentation of a profile expression fold change between the mutant and the control plant for all miRNAs. The provided range slider allows to quickly filter the records with a desired span of differentially expressed miRNAs. Additionally, the expression data for miRNA biogenesis mutants for any particular molecule can be accessed from the corresponding miRNA record.

**Fig. 2:**
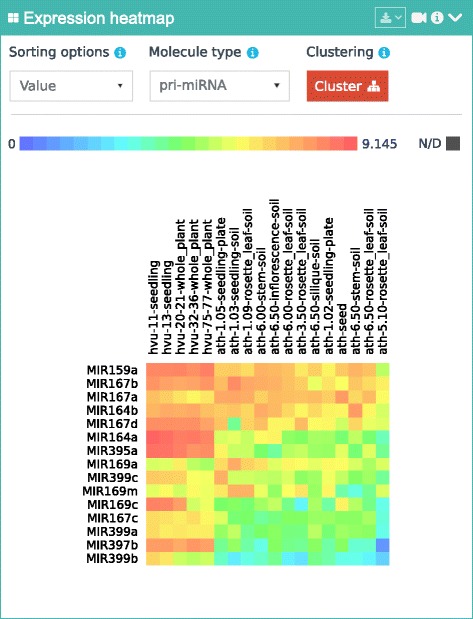
Example of a heat map-based display of multi-species comparison

The real potential of the mirEX 2.0 portal is based on its ability to combine different datasets into one display. There is no limit as to the number and type of selected plant species, developmental stages and microRNAs that can be combined in a single query. Depending on the complexity of the search, the interface determines the most user-friendly and informative way of presenting the results; for example, in addition to line and bar graph outputs, mirEX 2.0 offers a heat map-based display of multiple miRNA entries across complex (multi-tissue/multi-species) comparisons (Fig. 2). The new display functionalities allow for dynamic sorting of the expression level in a given tissue/species or for particular miRNA records as well as simple hierarchical clustering for data mining applications. Another visual feature of the profiling expression for large collections of miRNA records in a single stage/tissue is also provided in the form of a tag-based cloud where the level of expression is indicated by the color and size of the sequence ID. In this way the user can quickly identify the highest, lowest and similarly expressed miRNAs in the analyzed data set.

**Fig. 3:**
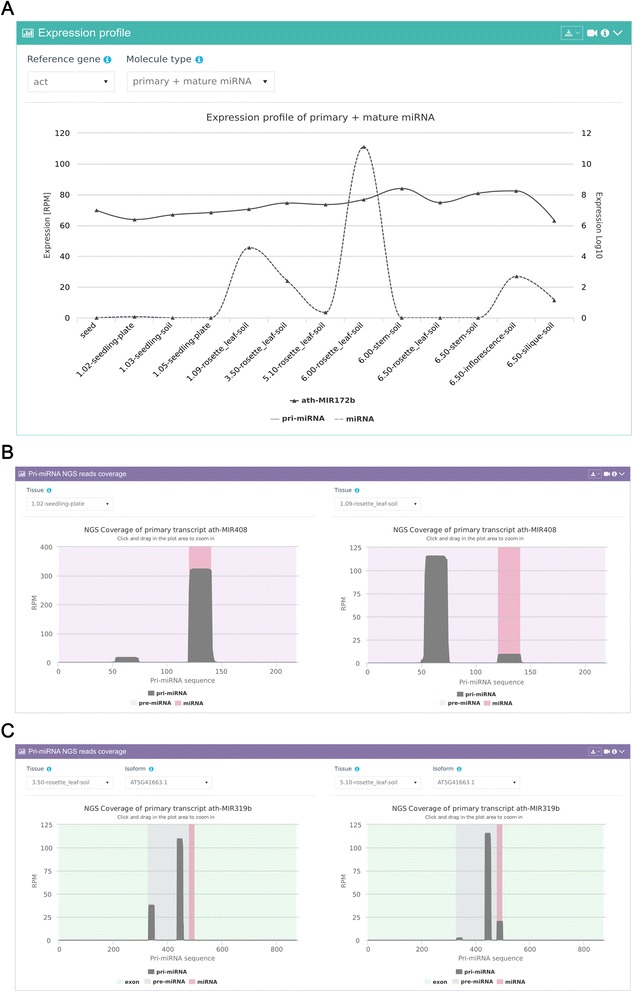
Representative visualizations of miRNA gene expression: **a** a line graph displaying combined expression of pri-miRNA (RT-qPCR) and mature microRNA (NGS) for miR172b. **b** The expression pattern of miR408/miR408* across various stages of arabidopsis development, and **c** the coverage pattern of sRNA fragments on the pri-miR319b transcript sequence

A key feature available in the mirEX 2.0 database is the integration of NGS sRNA-seq data into graphical expression plots along with the RT-qPCR-based transcript level measurements in the context of multiple developmental stages and tissues. This type of presentation provides a unique opportunity to simultaneously investigate expression levels for multiple miRNAs at different stages of processing (Fig. 3). The expression for RT-qPCR-analyzed pri-miRNAs is presented as a fold change value, and the accumulation of mature molecules is presented as RPM (reads per million) counts normalized to all miRNAs identified in the sample.

From the analysis of the expression data deposited in the mirEX 2.0 database it is clear that the relation between the level of a given pri-miRNA and its mature miRNA may be complex; for example, in the case of miR172b from arabidopsis (Fig. 3A) we observe modest variation in the expression of the pri-miRNA transcript across all of the tested stages and tissues when compared to the increased accumulation of mature molecules observed in the leaves and the inflorescence. Such differences in transcript accumulation can be the result of pri-miRNA transcription and maturation efficiency and/or miRNA stability within the RISC complex. The integration of the sRNA sequencing results with the miRNA precursor information allows to identify novel processing patterns of the pre-miRNA transcripts; for example, by using the tools built into the mirEX 2.0 interface a clear change can be identified between the processing efficiency of miR408/miR408* across various stages of arabidopsis development (Fig. 3B). The coverage pattern of sRNA on pri-miR319b (Fig. 3C) shows another example of the mirEX 2.0 data mining potential towards the exploration of complex processing patterns of plant microRNA precursors.

The single miRNA record window constitutes the central part of the mirEX 2.0 database. Among the basic data characterizing each miRNA molecule, e.g., hairpin pre-miRNA structure, Northern hybridizations, or external database links, it also includes: (i) a graphical presentation of the expression levels in all of the tested growth stages and tissues in the form of “Electronic Fluorescent Pictographs” [62], (ii) expression profiles in mutant plants (where applicable), (iii) mature miRNA NGS-based data with multiple sequence alignment, (iv) the exon-intron structure of the pri-miRNA transcripts with options to highlight and download all of the presented features, and (v) automatic retrieval of the most recent articles from PubMed.

### Web interface

The portal is accessed through a clean, intuitive web design which provides the user with an optimal viewing experience of the website’s features by adapting the web page layout to the operated device (e.g., tablets or desktop computers). A major point of emphasis of the mirEX 2.0 design has been to develop individual pages that are intuitive, thus giving the user freedom to focus on the biological query being addressed.

With this in mind, several user-centered solutions have been provided to enhance the general efficiency of the data mining experience. All results displayed in mirEX 2.0 are presented as separate, clearly labeled windows with the graphical elements providing contextual clues as well as mouse-over tooltips and short video tutorials with feature details. Users can adjust the amount of information that is present on the page to their needs, as any window can be minimized or expanded at any given moment. Depending on the data type, each of the windows may contain tools for data presentation, e.g., for sorting, filtering and changing the data source, thus allowing the user to generate customizable and integrated results. Other features that enhance the readability of the presented information include interactive charts as well as searchable and sortable table views.

Other general functionalities incorporated in the portal include a status message prompter presented at every query-building step and results screens. This widget informs the user about the current status of the request; for example, the status window will contain information about selected species, stages and the number of microRNAs, or it will show the progress of data loading. Through the ‘Comment’ feature, users can comment on any resource present in the database and start discussions. We encourage feedback from the bioscience community in terms of verifying the information we provide as well as obtaining relevant data either from ongoing research or from previous research, both published and unpublished.

### Future perspectives

We constantly continue to collect high-quality expression data for already incorporated plant species following new discoveries and annotations. In the future, we also plan to include data from other, not-well-established model organisms. The database schema as well as the user interface are now tuned to allow for the exploration of any number of combination of species/tissues/mutants and developmental stages. We also plan to incorporate microRNA expression profiles from plants exposed to various biotic and abiotic stresses. Work on drought and heat effects on the microtranscriptome in arabidopsis and barley is already under way. The currently established modular scheme of data presentation and wizard-like querying interface create an environment that guarantees further expansion toward the assimilation of new datasets and the development of novel visualization tools.

## Conclusions

The mirEX 2.0 web-based portal is a one-stop solution for the exploration of plant microRNA expression data covering mutants and three plant species representing scientifically (*Arabidopsis thaliana*), economically (*Hordeum vulgare*), and evolutionarily (*Pellia endiviifolia*) attractive research models. The provided user-friendly tools allow to explore expression data in any combination of species, tissues and developmental stages, thus leading to the rapid discovery and hypothesis-building of underlying relations and regulatory mechanisms. The developed technology also allows for unlimited further expansion of the data content and provides an environment for the design of novel tools following the needs of the plant community involved in the exploration of microRNA biology.

### Availability and requirements

Project name: mirEX 2.0

Project home pages: http://www.combio.pl/mirex

Operating system(s): Platform independent.

License: Not required.

Any restrictions to use by non-academics: None.

## Additional file

Additional file 1: Table S1. Comparison of database content, basic features and web interface between mirEX1 and mirEX2.

## Abbreviations

AGO: Argonaute protein
CBP: CAP BINDING PROTEIN
DCL1: DICER LIKE 1
ADP 1: ADENOSINE DIPHOSPHATE RIBOSE-RIBOZYLATION FACTOR 1
EF1-alpha: Elongation factor 1-alpha
gapdh: Glyceraldehyde-3-phosphate dehydrogenase C subunit 1
HST1: HASTY1
HUA1: HUA ENHANCER 1
HYL1: HYPONASTIC LEAVES 1
miRNA: microRNA
pri-miRNA: Primary transcript of miRNA
NGS: Next generation sequencing
RISC: RNA-induced silencing complex
RPM: Reads per million
SE: SERRATE
ubq10: Polyubiquitin 10
